# Polyanhydride Microcapsules Exhibiting a Sharp pH Transition at Physiological Conditions for Instantaneous Triggered Release

**DOI:** 10.1021/acs.langmuir.3c02708

**Published:** 2023-11-17

**Authors:** Viktor Eriksson, Leyla Beckerman, Erik Aerts, Markus Andersson Trojer, Lars Evenäs

**Affiliations:** † Department of Chemistry and Chemical Engineering, Chalmers University of Technology, 412 96 Gothenburg, Sweden; ‡ Department of Materials and Production, RISE Research Institutes of Sweden, 431 53 Mölndal, Sweden

## Abstract

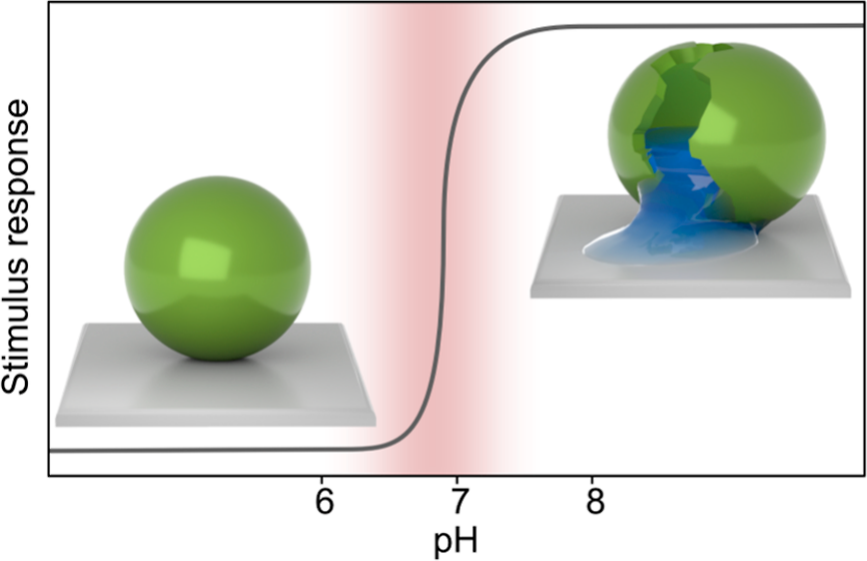

Stimulus-responsive microcapsules pose an opportunity to achieve controlled release of the entire load instantaneously upon exposure to an external stimulus. Core–shell microcapsules based on the polyanhydride poly(bis(2-carboxyphenyl)adipate) as a shell were formulated in this work to encapsulate the model active substance pyrene and enable a pH-controlled triggered release. A remarkably narrow triggering pH interval was found where a change in pH from 6.4 to 6.9 allowed for release of the entire core content within seconds. The degradation kinetics of the shell were measured by both spectrophotometric detection of degradation products and mass changes by quartz crystal microbalance with dissipation monitoring and were found to correlate excellently with diffusion coefficients fitted to release measurements at varying pH values. The microcapsules presented in this work allow for an almost instantaneous triggered release even under mild conditions, thanks to the designed core–shell morphology.

## Introduction

A controlled release of actives is often of key importance for product performance in several areas. Drug delivery in the pharmaceutical industry is the most common application for controlled release.^1−3^ However, controlled release is also important in other application areas such as self-healing materials,^4,5^ antifouling coatings,^6^ and food additives.^7^ Encapsulation of the actives into microcapsules is a promising route in all these areas for achieving not only a controlled release but also as a means of protecting sensitive actives. The concept of controlled release can often be narrowed down into two main categories with respect to the release mechanism: slow sustained release, which is driven by, e.g., diffusion or erosion, and fast triggered release that is activated by external stimuli.^8,9^ For triggered release, there are several chemical (pH, salinity), physical (radiation, heat, magnetization), mechanical (stress), and biological (host–ligand interactions) activation mechanisms that have been explored and innovated with typical release times on the order of minutes to hours.^9^ In medical applications, a pH-triggered release is convenient for, e.g., targeting the delivery of pharmaceuticals to specific regions of the gastrointestinal tract based on differences in local pH values.^10^ In wound care, a pH-triggered release could be utilized for the selective delivery of antimicrobial agents or growth factors to chronic wounds. Since bacterial colonization is one of the main factors behind the delayed healing of chronic wounds, antimicrobial substances are normally administered to prevent the formation of bacterial biofilms.^11^ Healthy skin normally shows a slightly acidic pH, whereas the pH instead is slightly alkaline in chronic wounds^12,13^ and this pH discrepancy could be utilized for the selective delivery of actives.

While these triggered release systems—responsive to a multitude of stimuli—are common in the literature, immediate release on the time scale of seconds is more uncommon albeit of importance for, e.g., precision in delivery. Focusing on the pH-triggered activation mechanism, at least one of the three main attributes is commonly sought in the microcapsules: a pH dependence in the solubility of the microcapsule barrier,^14,15^ a pH-dependent interaction between the active and the barrier,^16^ or pH-dependent chemical decomposition of the barrier.^17,18^ We have previously shown the potential of achieving instantaneous release by UV light-triggered decomposition of a polyphthalaldehyde shell.^19^ Polyanhydrides are a group of polymers showing pH-dependent hydrolysis of their anhydride groups along the backbone, leading to the possibility of achieving fast and complete pH-triggerable depolymerization.^20,21^ Uhrich and co-workers have extensively studied the synthesis and degradation kinetics of polyanhydrides copolymerized from salicylic acid and aliphatic diacids.^22−25^ Their work has shown that the labile anhydride linkage between the salicylic acid groups is stable at acidic pH, but its hydrolysis is greatly accelerated under slightly alkaline conditions. Most development toward applications for this class of polymers has been focused on macroscopic implantable devices or solid homogeneous microspheres, leading to comparatively long release time frames of at least several days.^26−28^ Recent work on polyanhydrides has focused on the synthesis of novel polyanhydride chemistries, however, with formulation still focused on sustained release over days to weeks from monolithic micro- or nanoparticles.^29−31^ To design a microcapsule system capable of achieving rapid triggered release, microcapsules of core–shell morphology are often advantageous.^19^ By dissolving the payload in the microcapsule core (either a liquid oil or water), only a thin solid polymeric shell in the range of hundreds of nanometers is required to encapsulate and protect it. This way, only the thin polymeric barrier must be ruptured to achieve a complete release of the active. This can be put in contrast to a solid microsphere, where the entire particle—often tens of micrometers—must disintegrate before releasing all active particles after exposure to the triggering event. In this work, we have used a polyanhydride, copolymerized from salicylic and adipic acid, to achieve an almost instantaneous triggered release at neutral to alkaline pH by formulating microcapsules of a core–shell morphology. The formulation in this work followed the route of internal phase separation^32^ to produce microcapsules containing oil cores. However, several formulation routes, applicable to the polyanhydride in this work, can be found in the literature to produce aqueous-core microcapsules.^33,34^ The connection between the release rate of actives and degradation kinetics of the polyanhydride shell has furthermore been studied in closer detail. Thus, both analytical determination of released degradation products from the microcapsule formulations and measurements on thin polyanhydride films by a quartz crystal microbalance with dissipation monitoring (QCM-D) were performed. These results were further correlated to in situ observations of the degrading capsules by microscopy.

## Experimental Section

The following chemicals were purchased from Sigma-Aldrich: Brij L23, chloroform (99.8%), dichloromethane (99.9%), ethyl linoleate (99%), phosphoric acid (85%), pyrene (99.0%), and sodium phosphate (monobasic and dibasic, 99%). The bis(2-carboxyphenyl) adipate polyanhydride (*M*_n_ ≈ 5.8 kDa) was from Polymer Source, acetone (99.8%) and sodium hydroxide (97%) were from VWR, poly(vinyl alcohol) (PVA, 100 kDa, 95% hydrolyzed) was from Acros Organics, and ethanol (99.5%) was from Solveco. All chemicals were used as received and without further purification. All water used was of Milli-Q quality (resistivity ≥18 MΩ cm).

### Microcapsule Formulation

To formulate the microcapsules in this work, a modified method based on internal phase separation by Loxley and Vincent was used.^19,32^ In short, the polyanhydride (93 or 108 mg), ethyl linoleate (37 or 22 mg), and pyrene (5% of the ethyl linoleate mass) were dissolved in a solvent mixture of 2.4 mL of dichloromethane and 200 μL acetone. This oil phase was dispersed into 3 mL of a 1 wt % aqueous PVA solution under high-speed shearing at 4000 rpm from a Kinematica Polytron PT3100D immersion dispenser equipped with a PT-DA07/EC-F101 dispersing aggregate. After 80 min of homogenization, the formed emulsion was diluted with an additional 3 mL of the aqueous phase and left under gentle magnetic stirring overnight for evaporation of the volatile solvents. Further studies were performed immediately after solvent evaporation to avoid excessive microcapsule degradation.

### Microcapsule Characterization

Microscopy was used to study and characterize the microcapsules in the aqueous suspensions. A ZEISS Axio Imager Z2m microscope was used with brightfield, differential interference contrast, and fluorescence illumination. For the fluorescence micrographs, pyrene was detected with filter set 49 (blue). The polyanhydride displayed autofluorescence from the salicylic acid moiety, which was seen by filter set 38HE (green). This allowed for the creation of composite micrographs, where the morphology could be deduced by differentiating between the oil core (blue) and polyanhydride shell (green).

From brightfield micrographs, the size distributions of the formulated microcapsules were also determined as previously described by us.^19^ In short, a semiautomated approach was employed in ImageJ (National Institute of Health) to analyze the size of at least 200 individual microcapsules in each sample. Following this, log-normal size distributions were fitted to the experimental data.

### Release Measurements

The release of pyrene from the microcapsules was quantified by transferring a small volume of the formulated microcapsule suspension (2 wt % microcapsules) to a larger volume of 100 mM phosphate buffer at pH values ranging between 2.2 and 12.5 under gentle magnetic stirring. Additionally, the nonionic surfactant Brij L23 (6 wt %) was also added to the release media to solubilize pyrene and ensure sink conditions. Aliquots were taken from the release medium at given times and filtered through 0.2 μm PTFE syringe filters to separate the microcapsules from the continuous phase. The released amount of pyrene in the continuous phase was then determined by UV–visible spectrophotometry using an HP 8453 spectrophotometer at 337 nm. To determine the total loading in the microcapsules, an aliquot of the release medium was taken out and diluted with three parts of ethanol to extract all pyrene. After at least 12 h of extraction, the samples were filtered and the concentrations were determined similarly by UV–vis spectrophotometry.

#### Diffusion Model

To evaluate the release of pyrene, a model based on Fickian diffusion^35^ was fitted to the experimental data as described previously by us.^36^ In short, the model describes Fickian diffusion in homogeneous spherical particles. Since the particles in this work were of a core–shell morphology rather than monolithic spheres, the model here described an apparent diffusivity in the multicompartment particles.^36^

### Degradation

#### UV–Vis Spectroscopy

Similar to the release of pyrene, the release of soluble polyanhydride degradation products from microcapsules into the aqueous release medium—as an indication of the microcapsule degradation rate at different pH values—was monitored over time by UV–vis spectrophotometry at 278 nm. The release of degradation products from both pyrene-loaded and empty microcapsules was studied. For pyrene-loaded microcapsules, the interference by the pyrene signal was minimized by subtracting a normalized pyrene reference spectrum from the recorded spectrum.

#### QCM-D

The degradation kinetics of the polyanhydride was also quantified on thin films by using a QCM-D E4 instrument (Biolin Scientific). Before measurements, the sensors were cleaned by immersion in dichloromethane for 30 min, after which they were dried with N_2_ gas. Following this, they were cleaned in a UV/ozone chamber for 30 min and then immersed in a base piranha (6:1:1; water/NH_3_/H_2_O_2_) for 5 min at 65 °C. Finally, the sensors were dried again with N_2_ gas. Immediately after cleaning, thin polyanhydride films were coated on the sensors in a SPIN 150 spin coater (SPS-Europe) using 50 μL of a 1 wt % polyanhydride solution in chloroform. The sensors were mounted on a Teflon support and then spin-coated at 2000 rpm for 60 s.

The thickness of the coated film was determined by measuring the frequency shift between the freshly cleaned and spin-coated sensor. Using the Sauerbrey equation,^37^
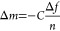
1the mass change, Δ*m*, could be related to the frequency change, Δ*f*, for a given overtone *n* (*n* = 1, 3, 5, 7, ...) by the sensor-dependent mass sensitivity constant *C*. The film must be rigid for eq 1 to be valid, and this was experimentally determined by also monitoring the energy dissipation from the crystal^38^ in addition to the frequency change. Similarly, the mass change from polymer degradation was determined using eq 1 by measuring the frequency change of the coated sensor as phosphate buffers of varying pH values flowed over the sensor at a rate of 100 μL/min. Here, the nonionic surfactant was omitted in the aqueous media to avoid interference from surfactant adsorption onto the sensors. For measurements at pH 12.5, a solution of pH 2.2 initially flowed over the surface for at least 60 min to obtain a baseline that was as stable as possible.

## Results and Discussion

### Formulation and Characterization of Microcapsules

**Figure 1:**
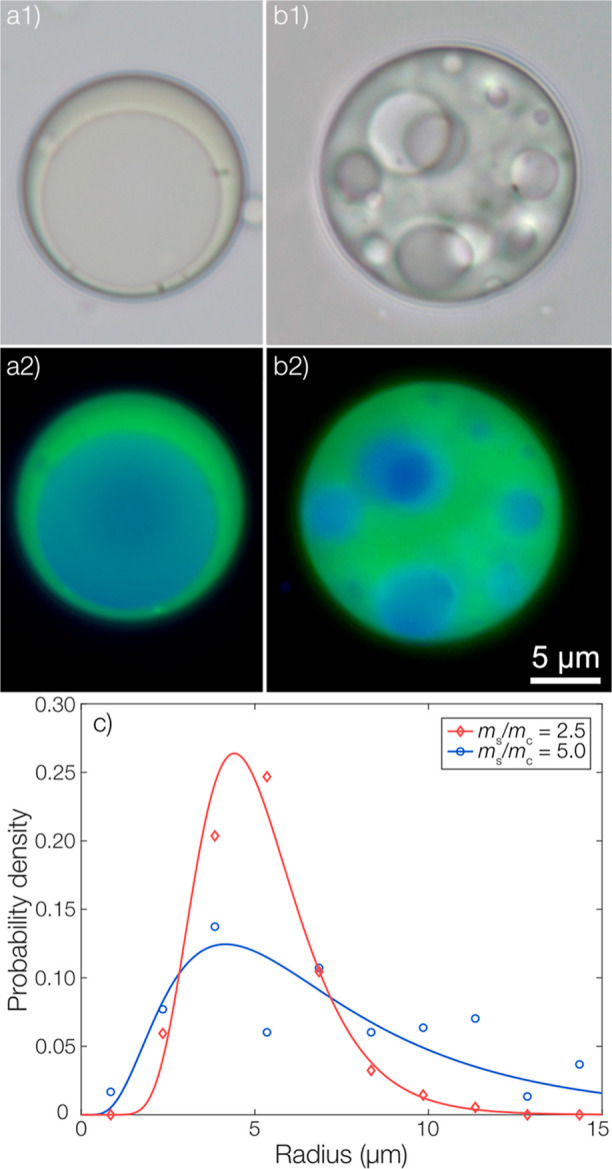
Micrographs of microcapsules with an *m*_s_/*m*_c_ of (a) 2.5 and (b) 5.0. Images in (a1) and (b1) are visualized by brightfield illumination, whereas (a2) and (b2) show overlaid fluorescence micrographs of the autofluorescent polyanhydride shell (green) and the molecularly dissolved pyrene in the ethyl linoleate oil phase (blue). The scale bar is valid for all micrographs. (c) Size distributions of microcapsules with an *m*_s_/*m*_c_ of 2.5 and 5.0. Data points are shown along with fitted log-normal distributions.

The polyanhydride-ethyl linoleate core–shell microcapsules presented here were formulated following a modified route^19^ based on internal phase separation by solvent evaporation.^32^ To study the effect of different microcapsule morphologies on the release, the formulation was tuned to produce either single-core or multicore microcapsules. This was enabled through variation of the shell-to-core mass ratio (*m*_s_/*m*_c_) of the microcapsules. As can be seen in Figure 1a,b, the obtained morphologies differed depending on the fraction of shell-forming material in the microcapsules. At an *m*_s_/*m*_c_-ratio of 2.5, the capsules were of single-core morphology. Upon increasing the *m*_s_/*m*_c_ to 5, multicore microcapsules were observed instead. The origin of this morphological difference is kinetic in nature and will be the topic of another publication. A larger number of microcapsules of each type are shown in the Supporting Information, verifying the lack of morphological variation within each subset of microcapsules. Despite the difference in morphology when comparing the different *m*_s_/*m*_c_-ratios, the average sizes of the microcapsules were similar as shown by the size distributions from micrographs in Figure 1c. A slightly broader distribution was observed with an increasing shell fraction, possibly indicating an increased coalescence during formulation.

### Degradation Kinetics of the Polyanhydride Shell

**Figure 2:**
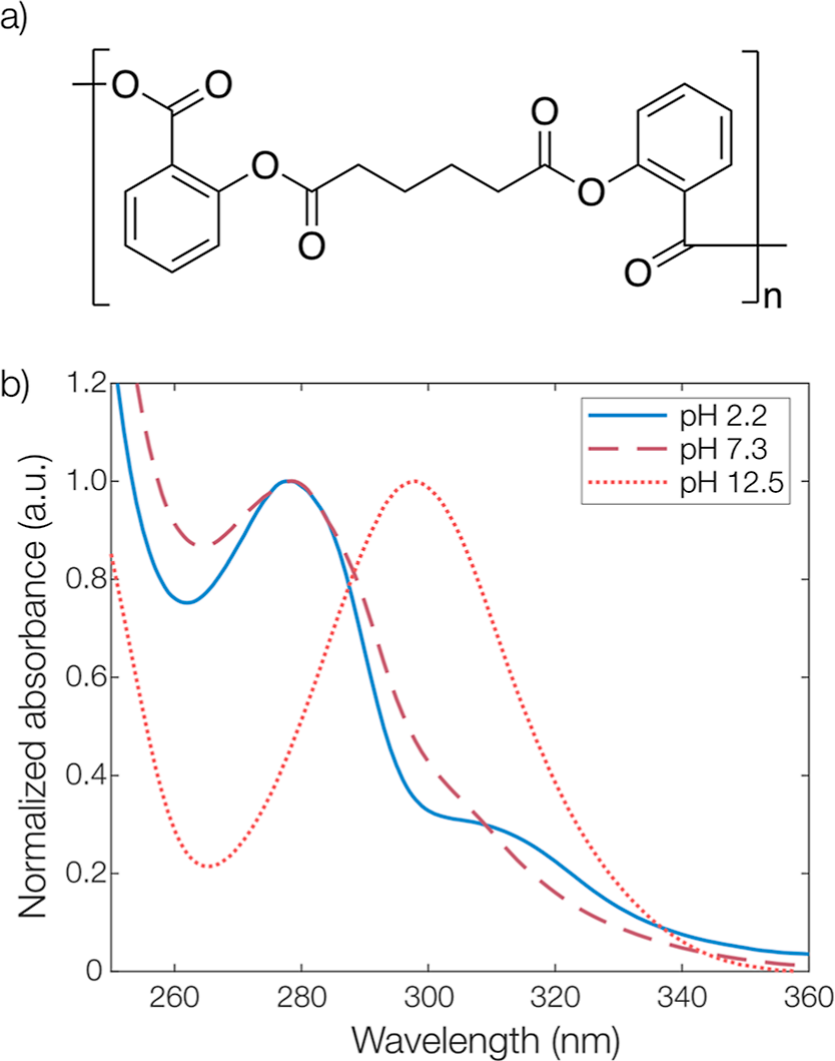
(a) Molecular structure of the poly(bis(2-carboxyphenyl)adipate) polyanhydride used in this work. (b) UV–vis absorption spectra of degradation products in pH 2.2 to 12.5. All spectra were normalized to the peak at either 278 or 298 nm.

Polyanhydrides such as the poly(bis(2-carboxyphenyl) adipate) shown in Figure 2a can undergo hydrolytic chain scission at both anhydride and ester bonds. Hydrolysis of the anhydride bond results in the formation of salicylic and adipic acid trimers, which—upon complete degradation—can be further broken down into their monomeric constituents by cleavage of the ester bonds. Uhrich and Erdmann^23,25^ have previously studied the degradation kinetics of such salicylate-based polyanhydrides in macroscopic, millimeter-sized devices. They showed that the anhydride group is highly sensitive to alkaline pH, while the ester group is much more stable.

In Figure 2b, representative UV–vis absorption spectra of the formed degradation products from microcapsules exposed to aqueous phosphate-buffered media for 1 h at varying pH values are shown. At pH 2.2 and 7.3, a maximum absorbance was found at 278 nm, which can be compared to the absorbance maximum of free salicylic acid at 296 nm. This was indicative of slow hydrolysis of the anhydride group at lower pH, with mostly salicylic-adipic acid trimers present in the solution. However, as indicated by the weak signal at around 300 nm in these samples, there was likely a low degree of ester hydrolysis in these samples as well. At pH 12.5, there was a shift in the maximum wavelength to 298 nm, indicating complete and fast hydrolysis of both anhydride and ester groups.^39^

Regarding the degradation kinetics, there is a key difference between the macroscopic materials studied by Uhrich and co-workers as mentioned above, and microcapsules with submicron shells.^20,40^ For sufficiently large and hydrophobic objects, water will only swell and hydrolyze the outermost surface, which leads to surface erosion of the material. However, for thinner materials, water will swell the entire matrix which leads to bulk erosion following pseudo-first-order degradation kinetics.^40^ A critical device dimension can be defined to differentiate between surface and bulk erosion, and this has been estimated to be on the order of 100 μm for polyanhydrides.^20^ The shells of the microcapsules, on the other hand, were on the order of hundreds of nanometers at their thinnest regions, estimated from micrographs. Therefore, bulk degradation of the microcapsule shells was assumed to occur.

To model the degradation from these submicron bulk-eroding matrices, we assumed water to be in excess and that there were no diffusion limitations during the degradation process which led to a pseudo-first-order rate equation for the mass *m*(*t*) remaining at time *t.*(41,42)
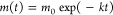
2

**Figure 3:**
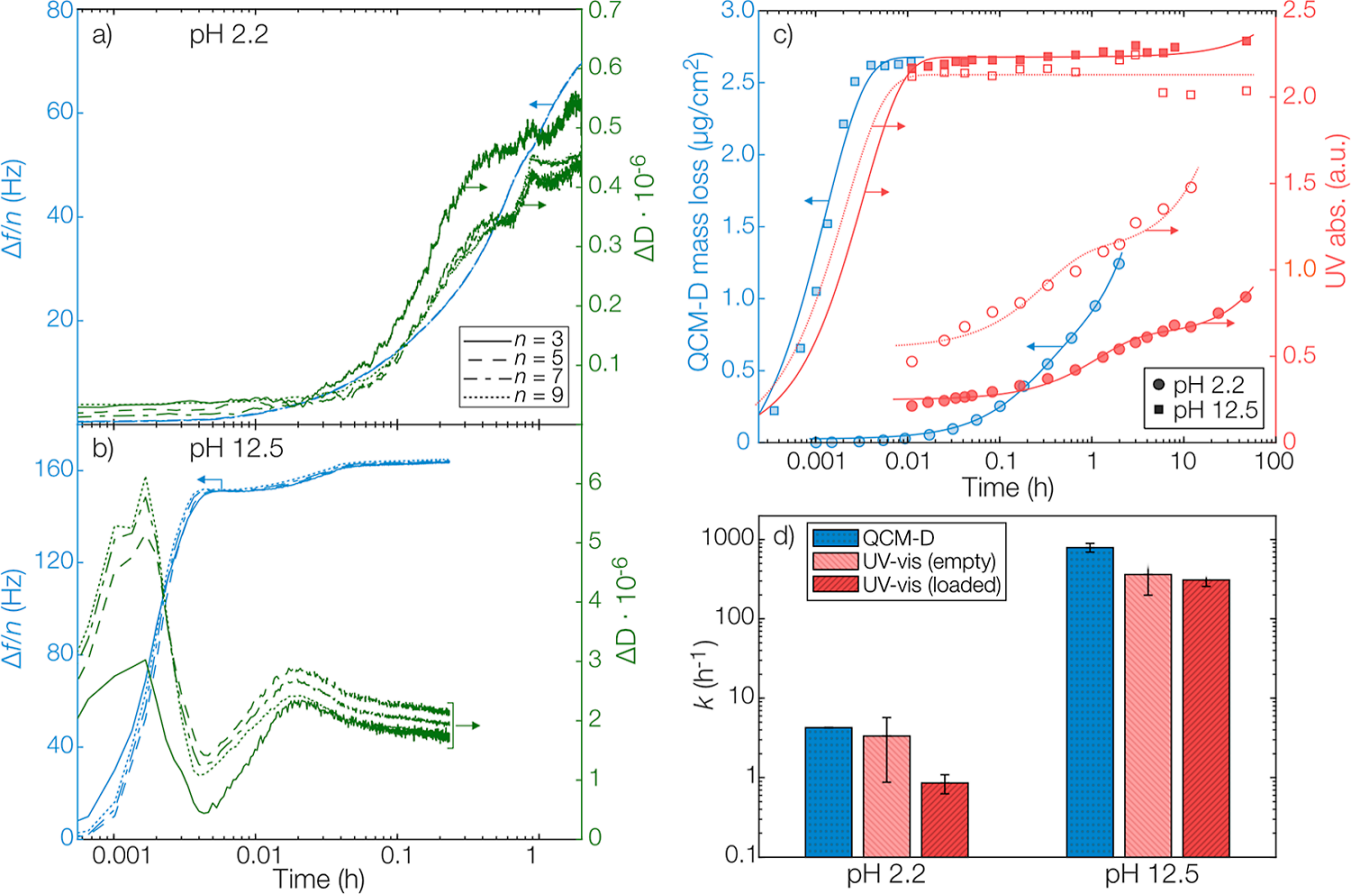
Frequency and dissipation changes during QCM-D measurements on thin polyanhydride films for overtones 3, 5, 7, and 9 at pH (a) 2.2 and (b) 12.5. (c) Polyanhydride degradation kinetics as studied by calculated mass loss from thin films using QCM-D (blue) and by released degradation products from microcapsules using UV–vis spectrophotometry (red) at pH 2.2 and pH 12.5. For QCM-D data, not all data points in parts (a,b) are shown to simplify visualization. The lines are fit to pseudo-first-order rate equations. Red-filled markers and solid lines represent data taken from pyrene-loaded microcapsules, and open markers and dotted lines represent data from empty microcapsules without pyrene. (d) Fitted degradation rate constants (±95% confidence interval of fit) based on spectrophotometry (red) on both empty and pyrene-loaded microcapsules and QCM-D data (blue).

Here, *m*_0_ is the initial mass and *k* is the rate constant for the degradation reaction. The model in eq 2 was fitted to experimental degradation data from both QCM-D and UV–vis spectroscopy (Figure 3c). In addition to the exponential decay, a linear baseline was applied to the data.

Microcapsules encapsulating the model active pyrene were used both for these degradation studies as well as for the triggered release measurements described below. The polymer degradation kinetics was investigated directly based on the spectrophotometric release data from microcapsules using UV–vis spectrophotometry. It was therefore necessary to subtract the pyrene signal from the UV–vis absorption spectra to yield a time-resolved absorbance of degradation products from which degradation rate constants could be determined as shown in Figure 3d. The validity of this procedure was confirmed by measurements on empty reference capsules (containing no pyrene) at selected pH values (Figure 3c). For both empty and pyrene-loaded microcapsules, only lower limits for the rate constant could be determined at high pH values due to experimental sampling frequency limitations. In Figure 3c, only the end points of the investigated pH range are shown. The full series of time-resolved degradation data acquired from spectrophotometry is shown in Supporting Information, and the fitted degradation rate constants are discussed in more detail in connection to Figure 5.

To further quantify the details of the degradation kinetics and corroborate the results obtained using spectrophotometry—especially at high pH where the temporal resolution was limited by the spectrophotometric data sampling—QCM-D measurements on thin films with dimensions close to that of the microcapsule shells were conducted. The QCM-D measurements can, in addition to the degradation in terms of mass, provide insights into the viscoelastic and water sorption properties of the polymer film.^43^ The average mass of the spin-coated QCM-D films before degradation was determined as 7.4 ± 0.8 μg cm^–2^, corresponding to a thickness of approximately 60 nm. In Figure 3a,b, the changes in both resonance frequency (normalized to the overtone) and dissipation over time are shown at both pH 2.2 and 12.5. During the entire recorded degradation process, the relative dissipation was low which meant that the film could be considered rigid.^38^ However, a small increase in dissipation could be observed during the first 0.01 h (40 s) at pH 12.5. This was likely due to the rapid formation of monomeric or oligomeric fragments from the degradation that plasticized the film and increased its water uptake. Both factors increase the viscous contribution to the viscoelastic solid film.

Spectrophotometric and QCM-D data, along with applied rate models, are shown in Figure 3c and the obtained rate constants are presented in Figure 3d. As can be seen, eq 2 was well fitted for all data, and at least a 100-fold difference in reaction kinetics was found between the extremes of the investigated pH values. It should be noted that the degradation at pH 12.5 was faster than the temporal resolution of the experimental sampling for UV–vis spectrophotometry in the aqueous release medium. Already at the first measurement after 40 s, most of the microcapsules had been degraded. The fitted rate constant was therefore a lower boundary for the true rate constant that could be determined under the experimental conditions.

### pH-Triggered Release

**Figure 4:**
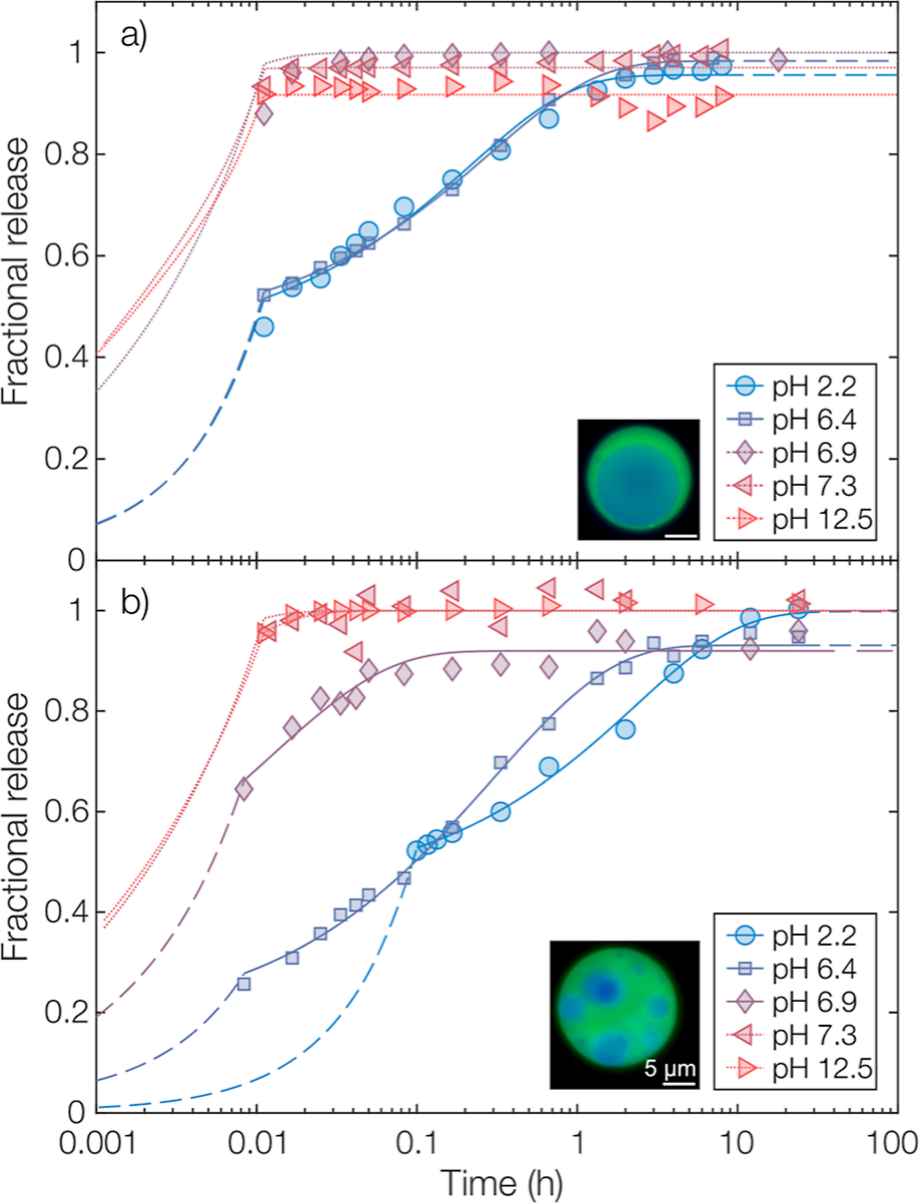
Fractional release from microcapsules with an *m*_s_/*m*_c_-ratio of (a) 2.5 and (b) 5.0, evaluated in release media with pH values ranging from 2.2 to 12.5. Experimental data points are shown along with fitted diffusion models as dashed and solid lines. Dotted lines are modeled threshold values for the apparent diffusivity given the temporal resolution of the experiments and are, consequently, only lower limits.

The pH-dependent release of the model substance pyrene into an aqueous phosphate-buffered release medium was studied, as shown in Figure 4. At low pH of 2.2 and 6.4, the release was slow and of sustained nature^36^ as expected for both *m*_s_/*m*_c_-ratios studied. This suggested that the rate-limiting step at low pH was the diffusion of pyrene through the microcapsule shells^8^ and not shell degradation. An apparent burst release could, however, be observed in these cases. This was a result of nonencapsulated oil droplets in the formulated microcapsule dispersion from which all of their dissolved pyrene would be rapidly solubilized in the release medium. When comparing the different shell-to-core ratios, a slower release was found for an increasing *m*_s_/*m*_c_-ratio despite the similarity in size of the different microcapsule types. For the diffusion-controlled release at lower pH values, the slower release was presumably caused by an increased diffusional path length. Although this was difficult to determine experimentally for the complex multicore morphologies from the micrographs in Figure 1b1,b2, it is reasonable to assume that the average shell thickness was greater with an increased *m*_s_/*m*_c_-ratio despite the change in morphology from single-core to multicore. Additionally, it could be observed that an increased *m*_s_/*m*_c_-ratio gave improved encapsulation with respect to the presence of free oil droplets.

Proceeding with the measurements at a higher pH of 6.9, this was the point of a rapid increase in the release rate. Above this critical pH value, the release was instantaneous within temporal resolution. This indicated a shift in the rate-limiting step from being diffusion-controlled to degradation-controlled. Here, a remarkably narrow interval in pH sensitivity could be observed. A change in pH from 6.4 to 6.9 resulted in a shift from sustained to an instantaneous and triggered release, showing the great potential for polyanhydride in a triggered release system. Again, different release profiles were observed for the different *m*_s_/*m*_c_-ratios. To explain this, the same reasoning as in the case of a lower pH could be used. Here, the slower release at increased shell thicknesses was instead likely due to a larger amount of shell material having to degrade before the core contents were exposed to the aqueous phase and released.

### The Sharp pH Transition

**Figure 5:**
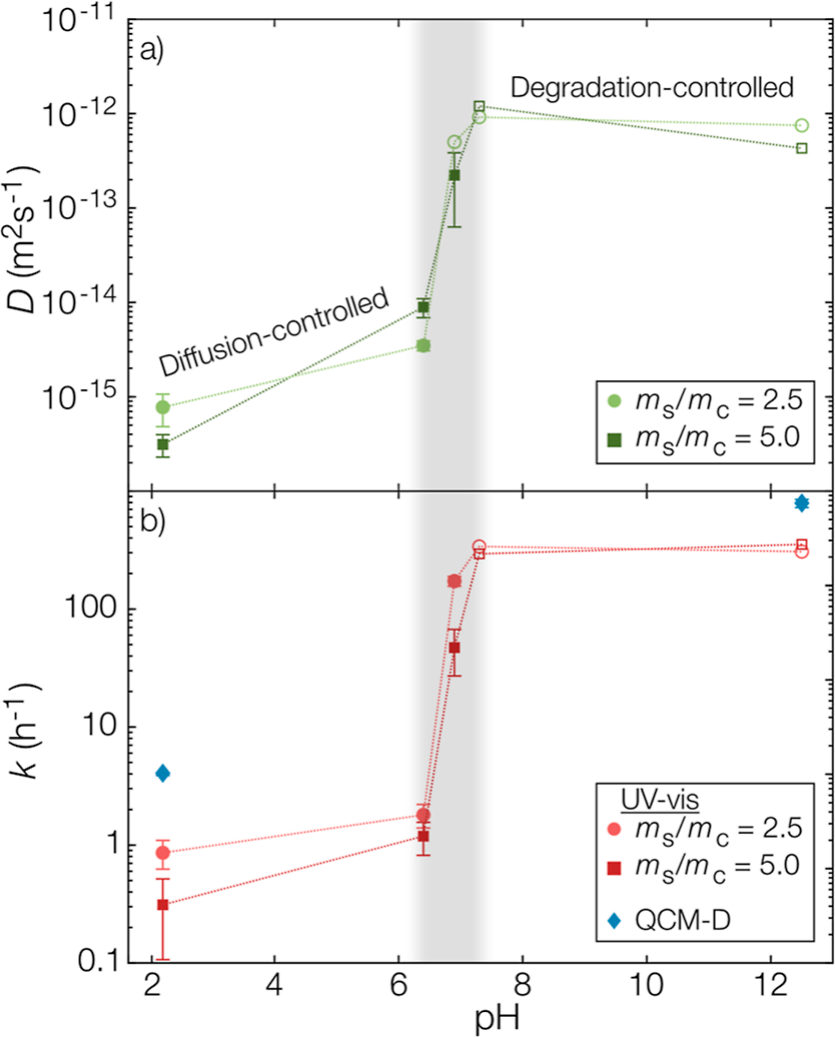
(a) Fitted diffusion coefficients (±95% confidence interval of fit) from microcapsules with an *m*_s_/*m*_c_-ratio of 2.5 and 5.0. (b) Fitted rate constants for the polyanhydride degradation based on spectrophotometry and QCM-D. All open markers in both subfigures are modeled threshold values given the temporal resolution of the experiments and, consequently, only lower limits.

To model the release data and quantitatively compare the measured release profiles, a simplistic Fickian diffusion model^35^ was employed. Here, the complex geometry of the formulated core–shell particles is simplified as monolithic spherical particles. This allowed for a quantitative comparison of the apparent pyrene diffusion coefficient between the different measurements. It must be emphasized that this was not the true diffusivity in the microcapsules since they contained two phases with significantly different physicochemical properties. Additionally, the diffusivity was assumed to be constant and independent of the time in each measurement. A gradually increasing diffusivity would probably be observed, because of the continuous degradation of the polyanhydride shell. This would result in the shell becoming more and more liquid-like and swollen by water throughout the process of degradation as indicated by the QCM-D studies, thereby increasing the pyrene diffusivity through the shell. When fitting this simplistic diffusion-based release model, apparent diffusivities on the order of 10^–16^ m^2^ s^–1^ were found at low pH values, see Figure 5. These values were larger as compared to those obtained for similar core–shell microcapsules prepared by us consisting of polylactide shells, where apparent diffusivities on the order of 10^–18^ m^2^ s^–1^ were found. This difference can be ascribed to the low chain length of the polyanhydride used here (5800 g mol^–1^) for which the flexible end groups start to affect the microporosity. We have previously shown that microporosity has a considerable impact on the release of actives.^44^

At the onset of the transition from diffusion-controlled release to degradation-controlled release, shaded in gray in Figure 5, a tremendous increase in the apparent diffusivity was observed. When comparing the increase in apparent pyrene diffusivity to the rate constant for polymer degradation (Figure 5b), an excellent agreement was found. A remarkable 100-fold increase in both apparent diffusivity and polyanhydride degradation rate constant could be observed over this narrow pH interval from 6.4 to 6.9. Again, it should be noted that the spectrophotometry-based data points in the degradation-controlled regime were only detection limits. Consequently, the QCM-D measurement at pH 12.5 with its improved temporal resolution was closer to the true value of the degradation kinetics. The full series of the time-resolved release of degradation products is shown in the Supporting Information.

### Morphological Weak Spots

**Figure 6:**
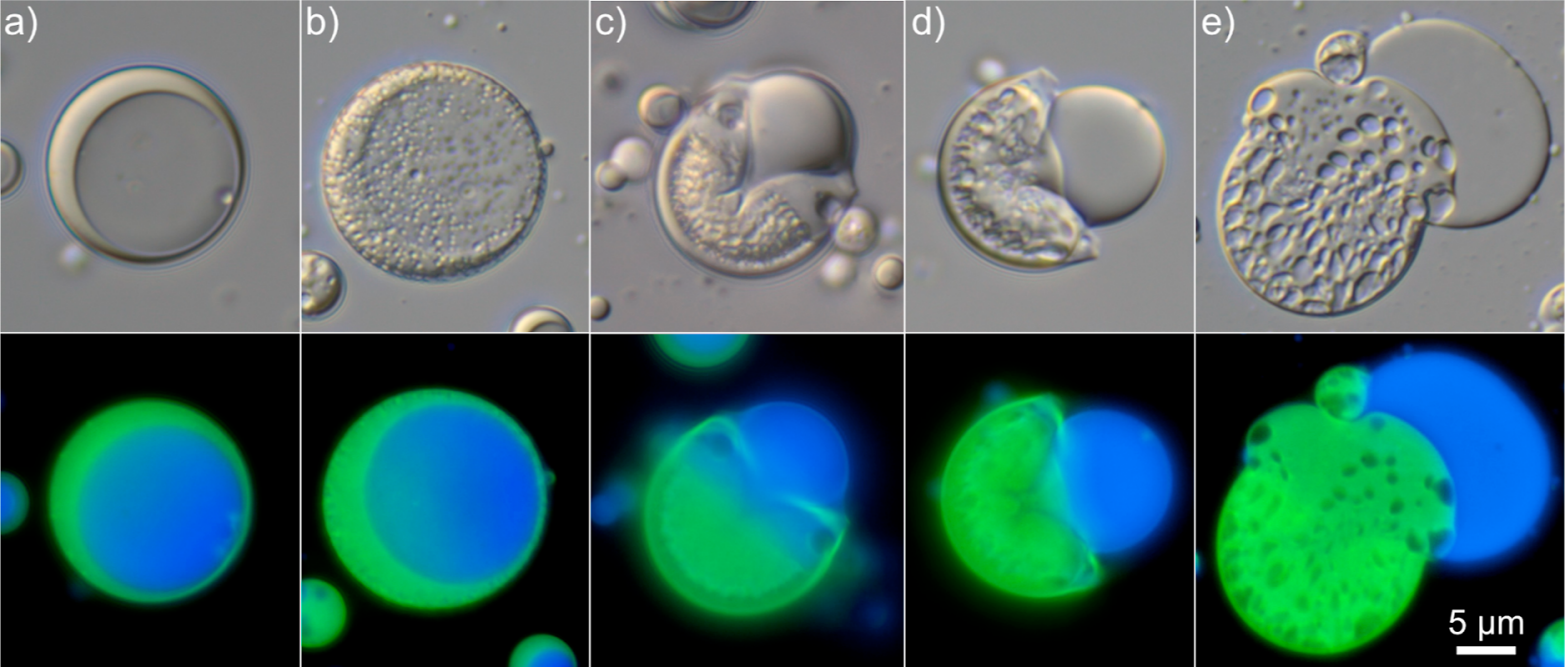
Morphological evolution over time as microcapsules (*m*_s_/*m*_c_ = 2.5) were exposed to an aqueous phase at pH 7.3. The appearance in (c) was seen within minutes of exposure, whereas (e) was after 20 min. Polyanhydride autofluorescence can be seen (green) and differentiated from pyrene dissolved in the ethyl linoleate core oil (blue). The scale bar is valid for all subfigures.

To gain a mechanistic understanding of the processes involved during microcapsule degradation and release, the capsules were studied in situ in the release medium over time, as shown in Figure 6. Within minutes of exposure to the release medium at pH 7.3 on a microscope slide, large-scale cracks could be observed in the microcapsule shells (Figure 6c) causing the entire core to leak out of the capsules. Following this, the disintegrating shells became more and more liquid-like as the shell degradation proceeded. This could be seen by the transformation from a hollow shell in Figure 6d to homogeneous droplets in Figure 6e after about 20 min of exposure. It should be noted that the solubilizing surfactant used in the release studies was omitted here to allow for better visualization of the different phases during the degradation process. The time scale for the observed degradation would likely be even faster in a well-stirred release medium compared to the stagnant conditions on the microscope slide.

A reasonable conjecture for the observed rapid pH-triggered response was that there was the presence of morphological weak spots. To be more precise, the oil core was not perfectly centered within the microcapsules but rather offset on one side, leading to an uneven shell thickness. As seen in Figure 6, the microcapsule clearly ruptured in the region where the shell was the thinnest to release its core contents. The discrepancy between the release profiles for the different *m*_s_/*m*_c_-ratios at the onset of degradation-controlled release (pH 6.9) may also be explained by this morphologically weak spot. Not all core material was within close proximity of the surface of the multicore capsules with an *m*_s_/*m*_c_-ratio of 5.0, meaning that a larger amount of the shell material had to degrade before exposing and releasing the core material. This probably led to a slower and more measurable release profile compared to the capsules with less shell material. Furthermore, there was a striking resemblance between the degradation mechanisms seen here and those observed previously by us^19^ for UV-light-triggered release polyphthalaldehyde core–shell microcapsules. For the latter, the immediate triggered release relied on a certain mechanically susceptible “blueberry” morphology. This further confirms the hypothesis of a weak morphological weak spot.

One benefit of using microcapsules of core–shell morphology as compared to the simpler and more frequently studied monolithic microspheres lies in the possibility of achieving an almost instantaneous triggered release, as previously mentioned. The system presented here is not dependent on fully degrading the polymer matrix to achieve a complete release. Instead, only a small hole must be formed in the shell, through which the oil core containing the active substance can be released. Therefore, the small pores that had formed in the capsule shell in Figure 6b within less than a minute of exposure may have already been large enough to facilitate the rapid release of the core contents.

## Conclusions

Polyanhydride microcapsules present an opportunity to achieve an almost instantaneous triggered release of actives under mild conditions. In this study, we investigated the degradation kinetics of polyanhydride core–shell microcapsules as well as thin films. At acidic pH, only minor degradation was found. However, an almost 100-fold increase in the degradation rate constant was found within the narrow pH interval of 6.4 to 6.9. This demonstrated remarkable responsiveness that could be used for delivery of, for example, antimicrobials to chronic wounds or drug delivery to specific regions of the gastrointestinal tract.

To utilize this pH responsiveness in a triggered release system, core–shell microcapsules were formulated. By studying the release in situ with a combination of optical and fluorescence microscopy, the release mechanism could be identified. Initially, pores appeared in the microcapsule shell, and within minutes, large cracks could be observed in the thinnest region of the shell, which exposed the core components to the surrounding medium. These findings were further found to correlate well with quantitative data on the release into well-stirred pH-buffered aqueous solutions. Over the narrow pH interval of 6.4 to 6.9, at least a 100-fold increase in apparent diffusivity of pyrene in the microcapsules was observed. This demonstrated the benefits of the chosen core–shell morphology of the microcapsules. Complete depolymerization of the shell was not necessary for a complete release of the entire payload—as would have been the case for a monolithic microsphere—but only a partial degradation to induce pores or cracks in the shell was necessary. Combined with the responsiveness of the chosen polyanhydride, this allowed for a complete release of the entire core within less than 1 min of exposure at neutral to slightly alkaline conditions.

## Supporting Information Available

Additional micrographs of microcapsules and time-resolved degradation data (PDF)
