# Supplementary material for: Polyanhydride Microcapsules Exhibiting a Sharp pH Transition at Physiological Conditions for Instantaneous Triggered Release
Source: Langmuir. 2023 Nov 17;39(49):18003–10. doi: 10.1021/acs.langmuir.3c02708 (PMC10720446; doi:10.1021/acs.langmuir.3c02708)
Supplement: Supplementary file 1 — la3c02708_si_001.pdf [file la3c02708_si_001.pdf]

**Suppporting Information**

# Polyanhydride Microcapsules Exhibiting a Sharp pH-transition at Physiological Conditions for Instantaneous Triggered Release

*Viktor Eriksson[a], Leyla Beckerman[a], Erik Aerts[a], Markus Andersson Trojer[b], and Lars Evenäs[a*]*

[a] Department of Chemistry and Chemical Engineering, Chalmers University of Technology, 412 96 Gothenburg, Sweden

[b] Department of Materials and Production, RISE Research Institutes of Sweden, 431 53 Mölndal, Sweden

# 1 Results

In Figure S1 micrographs visualizing the structure of microcapsules with an $m_s/m_c$ of 2.5 are shown. As can be seen, there is a polydispersity in size, although all microcapsules possess an offset core-shell morphology. From the fluorescence micrograph, a fraction of free oil droplets in the dispersion can be identified which are responsible for the burst release observed in the release measurements in the main article. In Figure S2, microcapsules with an $m_s/m_c$ of 5.0 are shown. These microcapsules were instead formulated to give an absolute majority of multicore microcapsules. Although both brightfield and fluorescence micrographs Figure S1 and Figure S2 depict the same region in the sample, slight translational movement was observed between each acquisition.

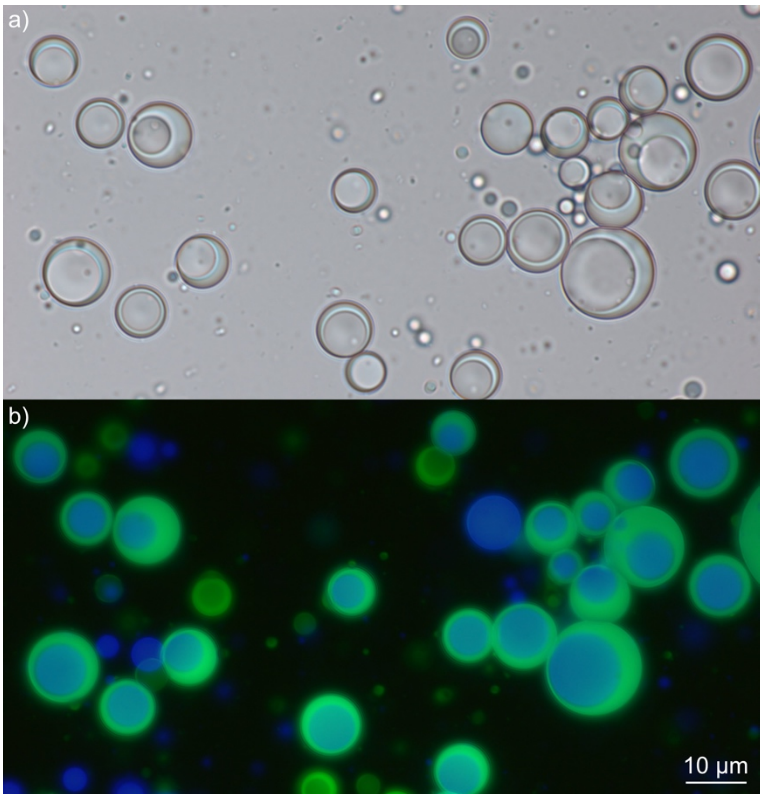


Figure S1. Micrographs of microcapsules with an $m_s/m_c$ of 2.5 visualized by a) brightfield illumination, and b) overlaid fluorescence micrographs of the autofluorescent polyanhydride shell (green) and the molecularly dissolved pyrene in the ethyl linoleate oil phase (blue). The scale bar is valid for both micrographs.

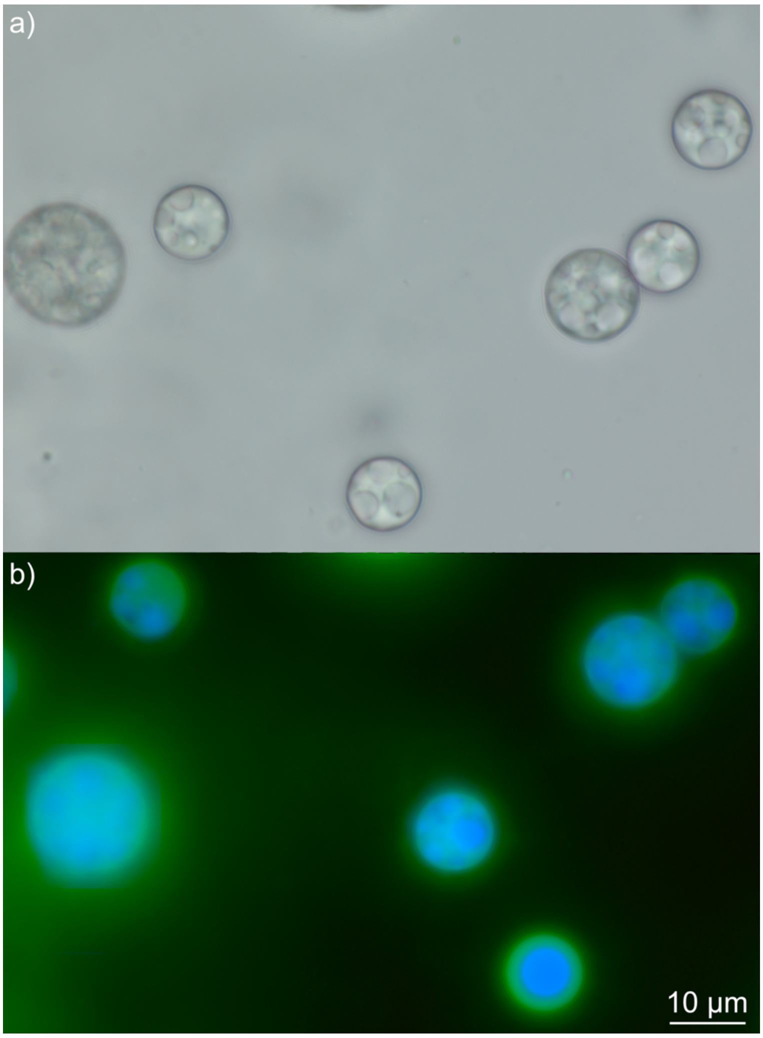


Figure S2. Micrographs of microcapsules with an $m_s/m_c$ of 5.0 visualized by a) brightfield illumination, and b) overlaid fluorescence micrographs of the autofluorescent polyanhydride shell (green) and the molecularly dissolved pyrene in the ethyl linoleate oil phase (blue). The scale bar is valid for both micrographs.

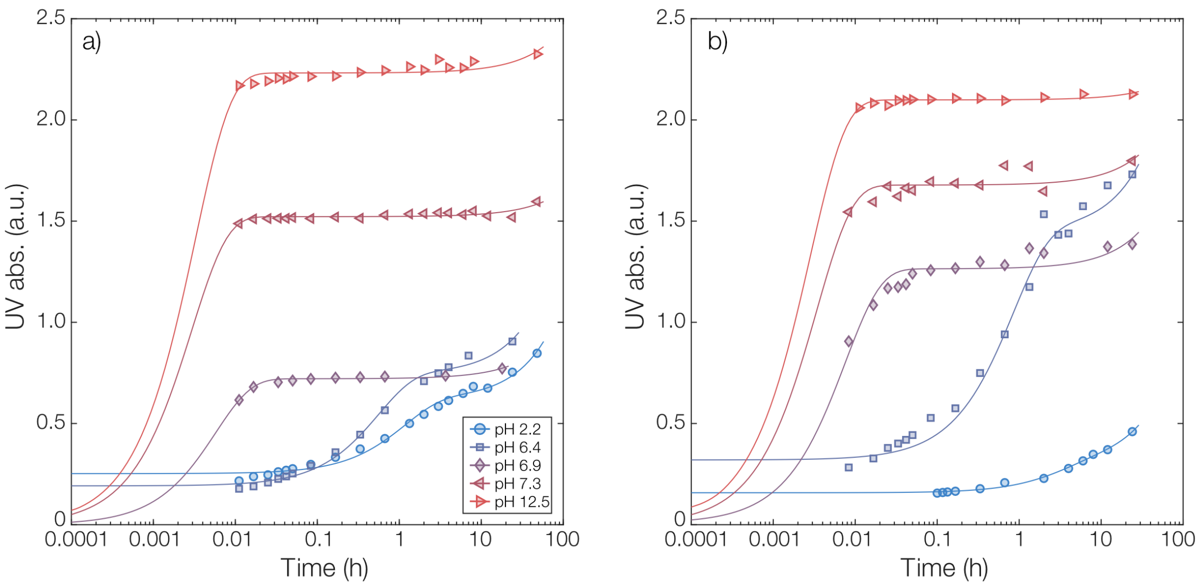


Figure S3. Time-resolved absorbance of polymer degradation products from microcapsules with an $m_s/m_c$ of a) 2.5 and b) 5.0 into release media at pH-values ranging from 2.2 to 12.5. The legend is valid for both subfigures.
